# Supplementary material for: First Report of Clonostachys rosea as a Mycoparasite on Sclerotinia sclerotiorum Causing Head Rot of Cabbage in India
Source: Plants (Basel). 2023 Jan 3;12(1):199. doi: 10.3390/plants12010199 (PMC9824872; doi:10.3390/plants12010199)
Supplement: Supplementary file 1 [file plants-12-00199-s001.zip › plants-1950864-supplementary.pdf]

**Supplementary files**

## Supplementary figures

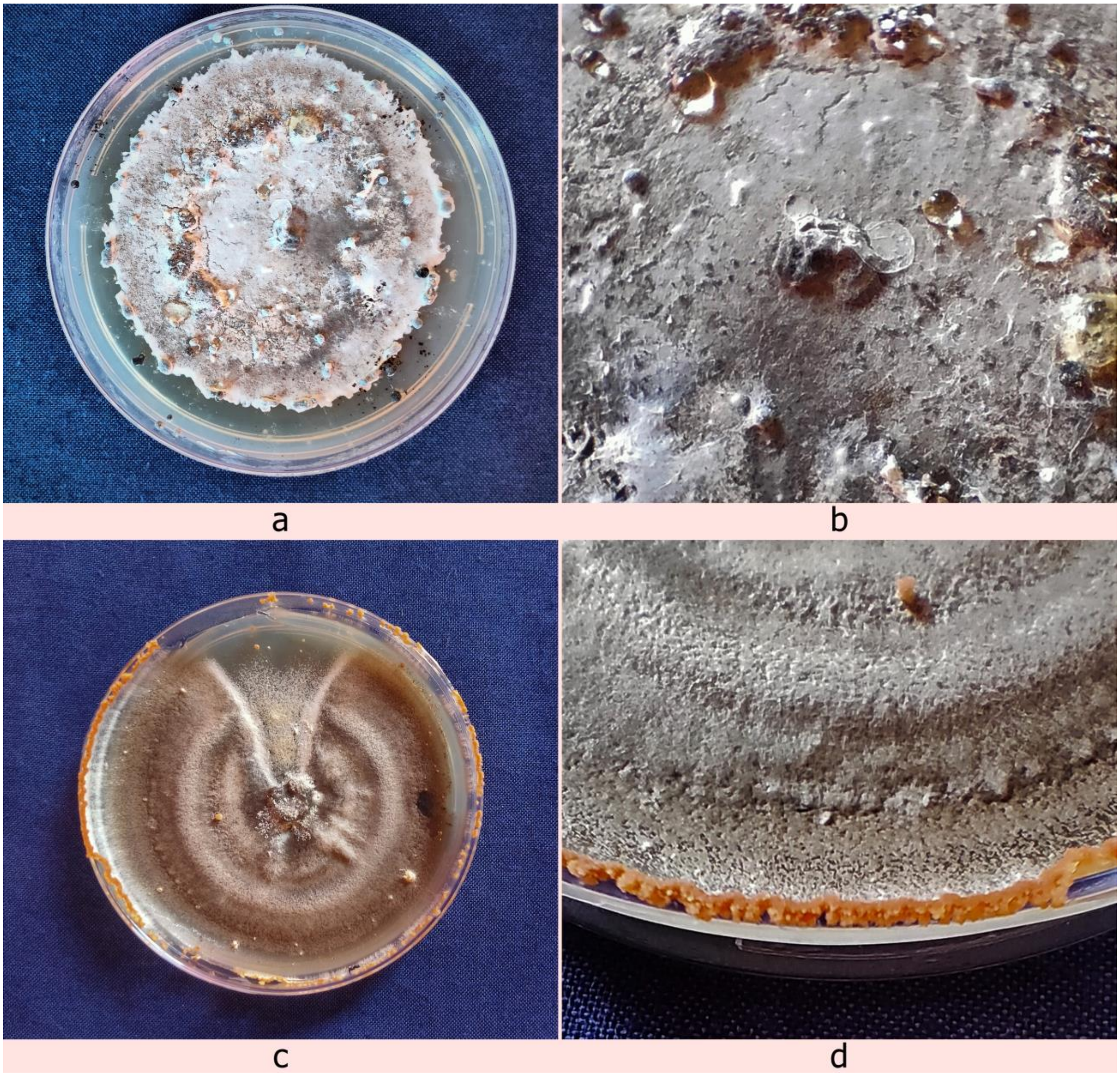


**Figure S1. Initial isolation of mycoparasitic fungi, *C. rosea* from infected sclerotia**

a- Mycoparasitized sclerotia placed in PDA medium
b- Presence of infection even in the secondary sclerotia
c- Gradual overgrowth of *C. rosea* growth from the infected sclerotia
d- Sporodochial growth over the plate

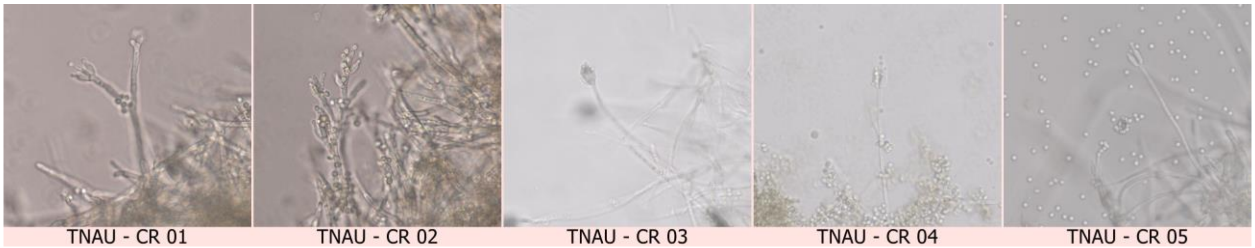


**Figure S2. Microscopic examination of fungal mycelium of *C. rosea* isolates under 40x magnification**

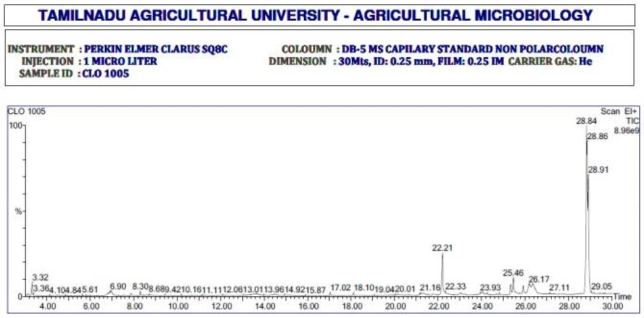


**Figure S3. Chromatogram showing the peaks of compounds in the fractions of *C. rosea* TNAU – CR 02**

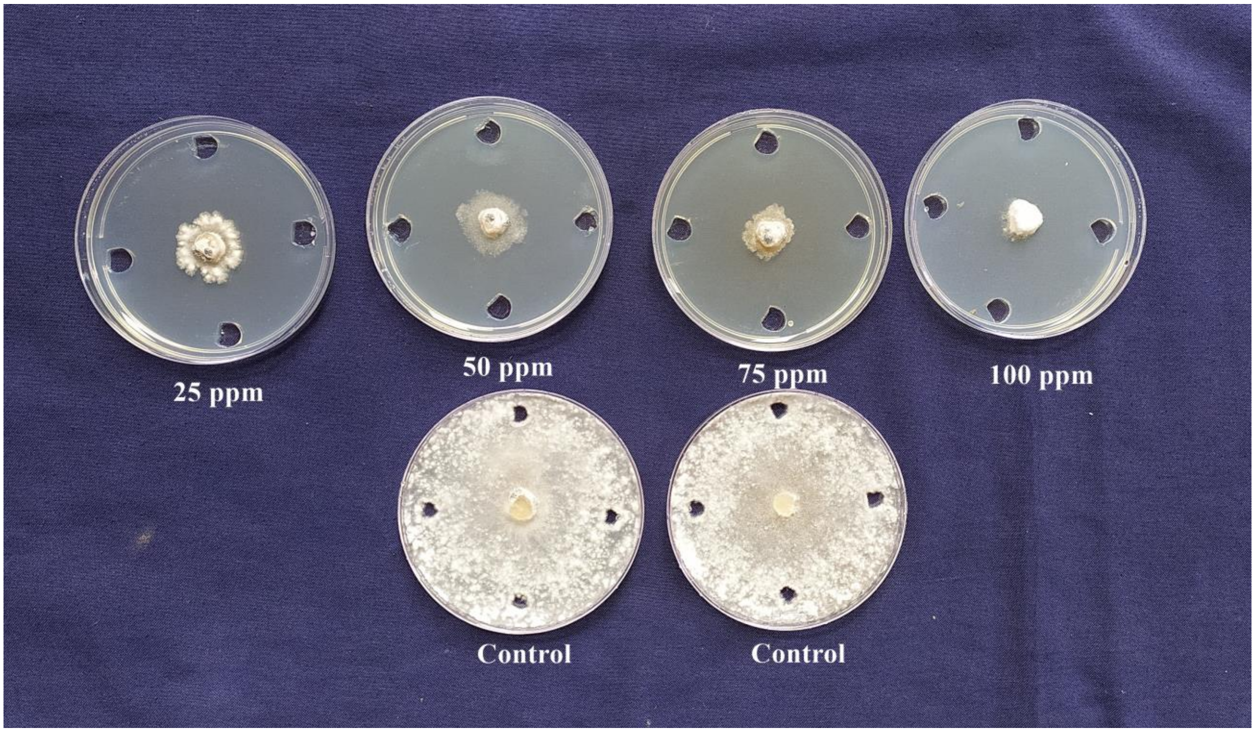


**Figure S4. Bioassay of crude antibiotics of *C. rosea* TNAU – CR 02 against *S. sclerotiorum***

**Supplementary table**

**Table S1. Colony morphology, pigmentation, and sporulation of C. *rosea* isolates**

| Isolate | Morphology | Pigmentation | Sporodochia coloration |
|---|---|---|---|
| TNAU – CR 01 | Whitish yellow sparse mycelium | Light yellow | Orange |
| TNAU – CR 02 | Slight yellow sparse mycelium | Light yellow | Orange |
| TNAU – CR 03 | White with light greyish mycelium | whitish yellow | Orange |
| TNAU – CR 04 | Dark Yellow dense mycelium | Dark yellow | Light Orange |
| TNAU – CR 05 | Whitish yellow sparse mycelium | Light yellow | Orange |

**Table S2. Bioassay of crude antibiotics of *C. rosea* TNAU – CR 02 against *S. sclerotiorum***

| Sl.No. | Concentration | Percent inhibition over control |
|---|---|---|
| 5 | 25 ppm | 82.17<br>(65.02)$^{b}$ |
| 6 | 50 ppm | 87.42<br>(69.22)$^{b}$ |
| 7 | 75 ppm | 92.33<br>(73.93)$^{a}$ |
| 8 | 100 ppm | 97.17<br>(80.32)$^{a}$ |

* Values are means of three replications,
Means in a column followed by same superscript letters are on par with each other according to LSD; Figures in parentheses represent arcsine transformation.
